# Mast cells in oral lichen planus and oral lichenoid lesions related to dental amalgam contact

**DOI:** 10.1590/1807-3107bor-2024.vol38.0005

**Published:** 2024-01-05

**Authors:** Mariana Saturnino de NORONHA, Giovanna Ribeiro SOUTO, Fernanda Aragão FELIX, Lucas Guimarães ABREU, Maria Cássia Ferreira AGUIAR, Elismauro Francisco MENDONÇA, Ricardo Alves MESQUITA

**Affiliations:** (a) Universidade Federal de Minas Gerais – UFMG, School of Dentistry, Department of Oral Surgery and Pathology, Belo Horizonte, MG, Brasil.; (b) Universidade Federal de Minas Gerais – UFMG, School of Dentistry, Department of Paediatric Dentistry and Orthodontics, Belo Horizonte, MG, Brasil.; (c) Universidade Federal de Goiás – UFG, School of Dentistry, Department of Stomatology (Oral Pathology), Goiânia, GO, Brazil.

**Keywords:** Mast Cells, Lichen Planus, Lichenoid Eruptions, Proto-Oncogene Proteins c-kit, Tryptases

## Abstract

The aim of this study was to analyze the expression of mast cell markers toluidine blue, c-kit, and tryptase and presence of mononuclear inflammatory cells in oral lichen planus (OLP) and oral lichenoid lesions related to dental amalgam. Nineteen specimens of OLP, OLLC, and healthy oral mucosa were selected. Mononuclear inflammatory cells were analyzed. Histochemical and immunohistochemical analyses were performed using toluidine blue, anti-c-kit and anti-tryptase reagents, and the results were quantified in areas A and B of connective tissue. Mast cells of all OLP and OLLC samples were positive for toluidine blue, c-kit, and tryptase. The density of toluidine blue+, c-kit+ and tryptase+ mast cells was higher in tissue with OLP and OLLC compared with healthy controls (p < 0.05). No difference was noted in mast cells density between OLP and OLLC (p > 0.05). The density of tryptase+ mast cells was higher in the subepithelial region (area A) than the region below it (Area B) in OLLC (p = 0.047). The mononuclear inflammatory cell density was higher in OLLC compared to OLP, but without statistical significance (p > 0.05). A positive statistical correlation was found between mononuclear immune cells and density of c-kit+ and tryptase+ mast cells in OLP (r = 0.943 and r = 0.886, respectively). Our data demonstrate that the etiopathogenesis process of OLP and OLLC modulates the expansion and degranulation of mast cells; mast cells density, however, was similar between OLP and OLLC. The distribution of mast cells appears to vary along the lamina propria.

## Introduction

Oral lichen planus (OLP), which affects 1% to 2% of the world population, is a mucocutaneous chronic inflammatory disease of the oral mucosa with or without simultaneous skin lesions.^1^ Although the etiopathogenesis of OLP remains unclear, it may be associated with an autoimmune dysfunction mediated by T-cells. Histopathological analysis of OLP typically shows a thickened ortho- or parakeratinized epithelium, band-like lymphocytic infiltration, and a disorganized layer of basal cells, similar to oral lichenoid lesions related to dental amalgam.^2^ OLLC are part of a type IV hypersensitivity reaction, typically associated with mercury and occurs in areas in contact with amalgam dental restorations.^3,4^ Despite some observed differences in the inflammatory infiltrate of OLP and OLLC, the clear differentiation between the two conditions is difficult.^2^ This is particularly important because both conditions are considered potentially malignant disorders with a malignant transformation rate of 1.37% for OLP and 2.43% for OLLC.^5^Therefore, the differential diagnosis of the two types of lesions based on clinical and histopathological characteristics remains challenging and relevant.^6^

Mast cells are granular cells of hematopoietic origin, usually involved in mucosal immune responses.^7^ Given their histological distribution, mast cells may be observed throughout the body, being a normal finding in mucosal tissues.^8^ Compelling evidence points out that mast cells play a broad and complex role in adaptive and innate immunity, including multiple nonspecific and specific stimuli in immune-mediated lesions, such as OLP and OLLC.^9,10^ Classically, mast cell activation synthesizes and releases granules with a spectrum of mediators, including histamine, proteases (tryptase, for instance), immunoregulatory cytokines and others inflammatory mediators.^7^ In turn, mast cell degranulation stimulates or inhibits neighboring cells.^11^

Mast cells are known to interact with other immune cells and these interactions are involved in several diseases, such as allergic responses in the airways,^12^ oral squamous cell carcinoma,^13^ and autoimmune diseases.^14^ The interaction between mast cells and T-cells seems to play a role in OLP’s immunopathogenesis.^15-17^ Equally, mast cells degranulation has also been associated with oral lichenoid lesions.^15,17-19^ Although heavy metals, mercury, gold, and silver can commonly induce hypersensitivity reactions in genetically susceptible individuals,^19,20^ the role of mast cells in subtypes of lichenoid lesions such as OLLC has not been fully explored.

Awareness of how mast cells engage in the etiopathogenesis of OLP and OLLC may guide the use of mast cell-stabilizing drugs and may be useful in their histopathological differentiation. We speculated that there are specific differences in mast cell activation and distribution along with differences in other mononuclear inflammatory cells between OLP and OLLC. To test these hypotheses, we investigated the density of mast cells by mast cell-specific surface markers, c-kit and tryptase, their degranulation in toluidine blue, and the density of mononuclear inflammatory cells in OLP and OLLC.

## Methodology

### Ethical issues

Our study was approved by the Ethics Committee of the Universidade Federal de Minas Gerais (UFMG, 2.361.404) and followed the principles for medical research set forth in the Declaration of Helsinki.

### Sample size calculation

Sample size, calculated based on data from a previous study comparing cell densities between OLP and OLLC^16^, was determined using the Power and Sample Size Calculation program (version 3.0, Nashville, USA). The previous data indicated a mean difference of 7.02 between groups and a pooled standard deviation of 5.16. Considering an alpha value of 0.05 and a statistical power of 80%, our study required at least six samples in each group to reject the null hypothesis that there is no difference in cell density between OLP and OLLC exists.

### Sampling

Paraffin-embedded tissues of lesions clinically and histopathologically diagnosed as OLP (n = 6) and OLLC (n = 7) were obtained from the archive of the Oral and Maxillofacial Pathology Service of UFMG. The inclusion criteria for OLP and OLLC were based on the American Academy of Oral and Maxillofacial Pathology criteria.^2^ There was no historical correlation with use of medication in patients with OLLC. Clinically, OLLC are distinguished from OLP by their close relationship to metal restorations, being locally and asymmetrically distributed.^2^ The histopathological inclusion criteria for OLP were the presence of band-like lymphocytic infiltration, a disorganized layer of basal cells, lymphocytic exocytosis, absence of oral epithelial dysplasia, and absence of verrucous epithelial alteration.^2^ Specimens of healthy oral mucosa (HOM) (n = 6) covering fully impacted third molars indicated for extraction were used as controls.

### Mononuclear inflammatory cell evaluation

Hematoxylin and eosin stained (H&E) slides were used to analyze the mononuclear inflammatory cells. The slides were scanned using the automatic digital slide scanner SlideViewer, 3DHistech, Carl Zeiss Vision GmbH, Gottingen, Germany). Then, the area of connective tissue infiltrated with mononuclear inflammatory cells in the scanned images was measured. Images obtained with SlideViewer® software also provided data that allowed manual counting of mononuclear inflammatory cells in the connective tissue at 40× magnification. The quantitative evaluation of cells via immunohistochemistry and toluidine blue staining was also performed with the use of the software. All cells were evaluated and counted by a previously trained observer (M.S.N.) at two different moments with a 15-day interval. The reliability of the measurements was assessed with the intraclass correlation coefficient (ICC; ICC > 0.91: very good correlation, 0.71 < ICC < 0.91: good correlation, 0.51 < ICC < 0.71: moderate correlation, 0.31 < ICC < 0.51: fair correlation, ICC < 0.31: poor correlation).^21^

### Toluidine blue and stain analysis

**Figure 1:**
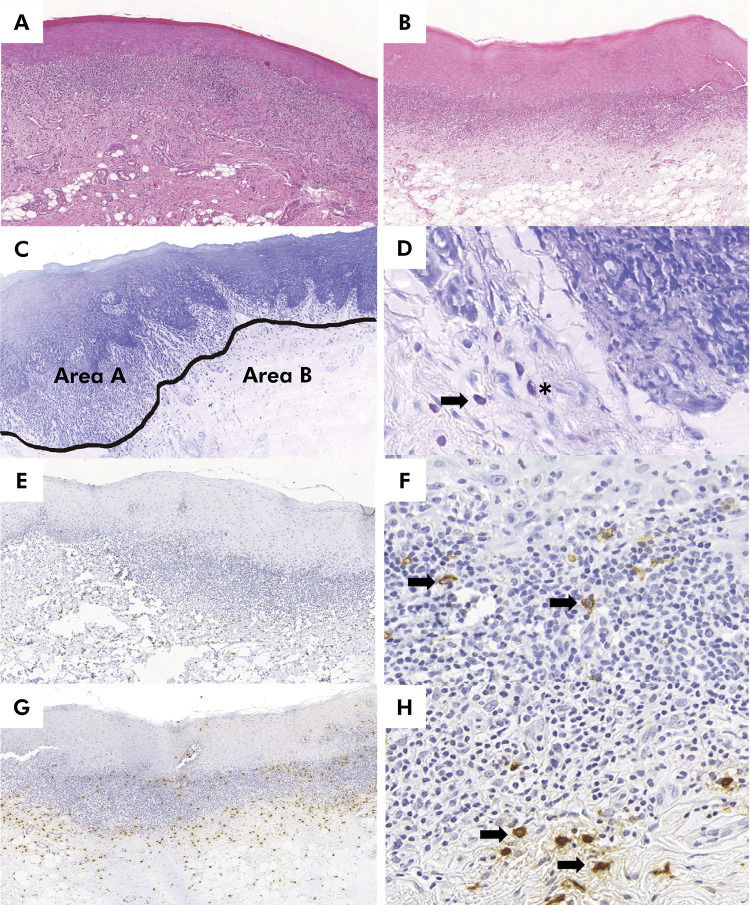
Histopathological, histochemical (toluidine blue), and immunohistochemical (c-kit and tryptase) features of oral lichen planus (OLP) and oral lichenoid lesions related to dental amalgam (OLLC). (A) OLP and (B) OLLC showing a well-defined band-like lymphocytic infiltrate below the epithelium (H&E, original magnification: 50×). (C) Tissue with OLLC and toluidine blue-stained showing evaluated areas, i.e, subepithelial band (area A) and region below the subepithelial band (area B) (toluidine blue, original magnification: 50×). (D) A non-degranulated mast cell is indicated by the arrow and a degranulated mast cell is indicated by the asterisk (toluidine blue, original magnification: 400×). (E) Immunohistochemical staining of c-kit in OLP (DAB, original magnification: 50×) and (F) in OLP showing mast cells (black arrow, DAB, original magnification: 200×). (G) Immunohistochemical tryptase staining in OLP and (H) in OLP showing mast cells (black arrow, DAB original magnification: 200×).

Four-µm-thick histopathological sections were obtained from paraffin blocks and deparaffinized, hydrated, and stained with 0.3% toluidine blue. The sections were washed, dehydrated, diaphanized, and framed with glass coverslips and Permount^®^ (Fisher Scientific, Fair Lawn, USA). Structures were stained by Toluidine blue and metacromasia was observed in granules of mast cells.^16^ The density of toluidine blue+ mast cells (Figure 1C), quantified in the connective tissue, was calculated according to the number of cells with clearly visible nuclei per mm^2^. More specifically, evaluations were performed in the region of the subepithelial band (i.e., area A) and below the subepithelial band (i.e., Area B). The toluidine blue+ mast cells were categorized in two groups according to the intensity of metachromasia or stainability and/or granule extrusion (degranulated or intact mast cells; Figure 1D), according to the criteria of Ghalayani et al.^17^

### Immunohistochemical and immunostaining analysis

Two 3-µm-thick histopathological sections were obtained from paraffin blocks and used for anti-c-kit and anti-tryptase tests. The sections were deparaffinized and hydrated, followed by antibody treatment with a 10-mM citrate buffer (pH 6.0) for 30 min at 98 °C. Endogenous peroxidase activity was blocked by placing samples in 0.3% hydrogen peroxidase for 15 minutes. The procedure was repeated 15 minutes later. Afterwards, samples were incubated with antibodies against c-kit (Dako, Carpinteria, USA) and tryptase (Cell Marque, Hot Springs, USA) at 1:200 and 1:500 dilutions, respectively, for 1 h at room temperature. Avidin-biotin complex was detected by using the LSAB^®^2 System-HRP Peroxidase Kit (K0690, Dako, Carpinteria, USA, K0690) and liquid 3,3’-diaminobenzidine (DAB; D5637, Sigma Chemical, St. Louis, USA). Mayer’s hematoxylin was used for counterstaining. The slices were dehydrated, diaphanized, and framed with glass coverslips and Permount^®^ (Fisher Scientific, Fair Lawn, USA).

The analysis of c-kit and tryptase immunostaining in areas A and B was conducted quantitatively considering the full length of the connective tissue at 400x magnification. Cells exhibiting brown membrane or cytoplasm staining were defined as immunoreactive to c-kit and tryptase.^22,23^

### Statistical analysis

The Statistical Package for the Social Sciences (version 22.0, SPSS Inc., Armonk, USA) was used for statistical analysis. Data concerning participant sex and age (demographics) as well as the specimen’s origin (clinical data) were analyzed descriptively.

Comparisons of toluidine blue, c-kit, and tryptase mast cell staining (cells/mm^2^) (lamina propria) between/among OLP, OLLC, and HOM were performed. Comparison of toluidine blue, c-kit, and tryptase staining in mast cell density (cells/mm^2^) (Area A and Area B) between OLP and OLLC and comparison of mononuclear inflammatory cells density (cells/mm^2^) between OLP and OLLC were also performed. Nonparametric tests (Mann-Whitney U and Kruskal-Wallis) were used. Data are presented as medians and interquartile ranges (25%–75%). P-value < 0.05 was considered statistically significant.

The Spearman correlation test was used to determine correlations between mononuclear inflammatory cells and the markers. P-value < 0.05 was considered statistically significant.

## RESULTS

### Clinical and histopathological features of OLP and OLLC

Demographic and clinical data of participants who provided the specimens are shown in Table 1. The mean age in years of individuals was 63.1 (OLP), 48.6 (OLLC), and 25.5 (HOM). Histopathological features of OLP included the destruction of the basal cell layer, bandlike inflammatory infiltrate in the connective tissue, atrophic epithelium, and the absence of both oral epithelial dysplasia and verrucous epithelial alteration (Figure 1A). OLLC exhibited numerous features of OLP (Figure 1B) and lymphoid follicles within a dense lymphocytic infiltrate were noted on occasion. We did not observe differences in cell type between the inflammatory infiltrate in OLP and OLLC.

**Table 1:** Demographic and clinical characteristics of the sample according to lesion type.

Variable	Oral lichen planus (n = 6)	Oral lichenoid lesions related to dental amalgam (n = 7)	Healthy oral mucosa (n = 6)
Demographics			
Age (years)	63.1 ± 9.3*	48.6 ± 15.1*	25.5 ± 5.7*
Female:male ratio	01:02	06:01	02:01
Specimen’s origin			
Buccal mucosa, bilateral	100%	14.3%	-
Buccal mucosa, unilateral	-	71.4%	-
Gum	-	14.3%	-
Oral mucosa covering third molars	-	-	100%

*Data are given in mean and standard deviation.

### Histochemical and immunohistochemical findings

All cases of OLP and OLLC were positive for toluidine blue, c-kit, and tryptase, mainly in the perivascular regions of the subepithelial band and below the subepithelial band (area A and area B/lamina propria) (Figures 1C–1H). HOM demonstrated a limited focal staining for the three markers in perineural and perivascular regions. Histochemical and immunohistochemical findings in lamina propria are shown in Table 2. The densities of total toluidine blue+ mast cells (p = 0.005) and degranulated mast cells (p = 0.004) were higher in OLP than in HOM. However, there were no differences in densities of non-degranulated mast cells analyzed by toluidine blue among OLP, OLLC, and HOM (p = 0.084). The results of c-kit and tryptase showed a higher mast cell density in OLP (p < 0.05) and OLLC (p < 0.05) than in control.

**Table 2:** Comparison of toluidine blue, c-kit, and tryptase staining in mast cell density (cells/mm2) (lamina propria) among oral lichen planus, oral lichenoid lesions related to dental amalgam, and healthy oral mucosa.

Markers (Lamina propria)	Mast cell density (cells/mm^2^)			
	Oral lichen planus (n = 6)	Oral lichenoid lesions related to dental amalgama (n = 7)	Healthy oral mucosa (n = 6)	p-value

	Median (Q25-Q75)	Median (Q25-Q75)	Median (Q25-Q75)	
Toluidine blue				
Total mast cells	129.1 (84.8–175.2)^a^	70.7 (45.4–121.9)^ab^	24.1 (14.4–37.6)^b^	0.002
Degranulated mast cells	108.3 (69.7–137.3)^a^	54.2 (31.3–85.6)^ab^	17.3 (9.6–26.6)^b^	0.001
Non-degranulated mast cells	20.8 (11.3–33.1)^a^	14.8 (10.0–31.5)^a^	5.0 (2.7–16.4)^a^	0.084
c-kit	126.2 (114.2–148.2)^a^	95.5 (75.4–143.7)^a^	45.7 (21.9–56.0)^b^	0.001
Tryptase	126.4 (98.0–155.1)^a^	119.3 (82.3–142.4)^a^	41.3 (18.2–81.5)^b^	0.006

p-value = Kruskal-Wallis test; Significant at p < 0.05. In the post hoc test, different letters mean significant difference.

No significant differences were observed in mast cell density between OLP and OLLC in areas A and B, regardless of the marker used (Table 3). Notably, the density of tryptase+ mast cells was higher in the subepithelial band (area A) than below the subepithelial band (area B) in OLLC (medians = 212.5 and 104.2, respectively; p = 0.047).

**Table 3:** Comparison of toluidine blue, c-kit, and tryptase staining in mast cell density (cells/mm2) (area A and area B) between oral lichen planus and oral lichenoid lesions related to dental amalgam.

Markers	Mast cell density (cells/mm^2^)		
	Oral lichen planus (n = 6)	Oral lichenoid lesions related to dental amalgama (n = 7)	p-value

	Median (Q25-Q75)	Median (Q25-Q75)	
Toluidine blue			
Degranulated mast cells			
Area A	87.3 (34.9–196.2)	54.6 (35.8–100.4)	0.699
Area B	99.0 (56.2–159.9)	35.3 (23.4–98.3)	0.180
Non-degranulated mast cells			
Area A	14.7 (5.9–32.3)	12.2 (5.8–34.4)	0.937
Area B	26.6 (17.6–33.3)	19.0 (11.7–39.8)	0.485
c-kit			
Area A	183.6 (100.0–264.1)	172.2 (99.2–266.6)	0.805
Area B	162.8 (107.0–206.4)	176.4 (141.9–223.8)	0.534
Tryptase			
Area A	222.8 (182.5–278.0)	212.5 (140.0–380.6)	0.999
Area B	150.6 (66.8–187.2)	104.2 (71.6–202.4)	0.945

p-value = Mann Whitney test; Significant at p-value < 0.05.

The densities of mononuclear inflammatory cells in lamina propria (p = 0.093) of Area A (p = 0.093) and Area B (p = 0.240) of OLLC were higher, albeit not significant, than in OLP (Table 4).

**Table 4:** Comparison of mononuclear inflammatory cell density (cells/mm2) between oral lichen planus and oral lichenoid lesions related to dental amalgam.

Mononuclear inflammatory cells density (cells/mm^2^)			

Variable	Oral lichen planus (n = 6)	Oral lichenoid lesions related to dental amalgam (n = 7)	p-value
	Median (Q25-Q75)	Median (Q25-Q75)	
Total lamina propria	771.8 (649–884.8)	1083.7 (818.7–1730.6)	0.093
Area A	1797.2 (1345.9–2198.7)	3090.2 (1948.3–3492.2)	0.093
Area B	77.9 (43.1–140.5)	181.3 (54.2–275.2)	0.240

p-value = Mann Whitney test. Significant at p < 0.05.

### Correlation between mononuclear inflammatory cells and markers

Correlations between mononuclear inflammatory cells and markers in OLP are shown in Table 5. We found a strong, positive, and significant correlation between mononuclear inflammatory cells and c-kit+ mast cells (correlation coefficient = 0.943) and mononuclear inflammatory cells and tryptase+ mast cells (correlation coefficient = 0.886) in area B of OLP. Correlations between mononuclear inflammatory cells and markers in OLLC are shown in Table 6. We also found a strong, positive, and significant correlation between mononuclear inflammatory cells and non-degranulated blue toluidine+ mast cells in lamina propria (coefficient correlation = 0.900) and between mononuclear inflammatory cells and tryptase+ mast cells in area A (correlation coefficient = 0.943) in OLLC. On the other hand, a strong, negative, and significant correlation was found between mononuclear inflammatory cells and total blue toluidine+ mast cells (correlation coefficient = -0.900) and mononuclear inflammatory cells and degranulated blue toluidine+ mast cells (correlation coefficient = -0.900) in area B in OLLC.

**Table 5:** Correlation between mononuclear inflammatory cells and markers in oral lichen planus.

Oral lichen planus	Coefficient*	p-value
Lamina propria (Area A and Area B)		
mononuclear inflammatory cells versus c-kit+ mast cells	0.200	0.704
mononuclear inflammatory cells versus tryptase+ mast cells	0.200	0.704
mononuclear inflammatory cells versus total blue toluidine+ mast cells	0.600	0.208
mononuclear inflammatory cells versus degranulated blue toluidine+ mast cells	0.657	0.156
mononuclear inflammatory cells versus non-degranulated blue toluidine+ mast cells	0.371	0.468
Area A		
mononuclear inflammatory cells versus c-kit+ mast cells	0.319	0.538
mononuclear inflammatory cells versus tryptase+ mast cells	0.371	0.468
mononuclear inflammatory cells versus blue toluidine+ mast cells	0.486	0.329
mononuclear inflammatory cells versus total degranulated blue toluidine+ mast cells	0.543	0.266
mononuclear inflammatory cells versus non-degranulated blue toluidine+ mast cells	0.486	0.329
Area B		
mononuclear inflammatory cells versus c-kit+ mast cells	0.943	0.005
mononuclear inflammatory cells versus tryptase+ mast cells	0.886	0.019
mononuclear inflammatory cells versus total blue toluidine+ mast cells	0.486	0.329
mononuclear inflammatory cells versus degranulated blue toluidine+ mast cells	0.486	0.329
mononuclear inflammatory cells versus non-degranulated blue toluidine+ mast cells	0.600	0.208

*Spearman’s correlation coefficient.

**Table 6:** Correlation between mononuclear inflammatory cells and markers in oral lichenoid lesions related to dental amalgam.

Oral lichenoid lesions related to dental amalgam	Coefficient*	p-value
Lamina propria (Area A and Area B)		
mononuclear inflammatory cells versus c-kit+ mast cells	0.771	0.072
mononuclear inflammatory cells versus tryptase+ mast cells	0.429	0.397
mononuclear inflammatory cells versus total blue toluidine+ mast cells	0.400	0.505
mononuclear inflammatory cells versus degranulated blue toluidine+ mast cells	0.400	0.505
mononuclear inflammatory cells versus non-degranulated blue toluidine+ mast cells	0.900	0.037
Area A		
mononuclear inflammatory cells versus c-kit+ mast cells	-0.200	0.704
mononuclear inflammatory cells versus tryptase+ mast cells	0.943	0.005
mononuclear inflammatory cells versus total blue toluidine+ mast cells	-0.400	0.505
mononuclear inflammatory cells versus degranulated blue toluidine+ mast cells	-0.300	0.624
mononuclear inflammatory cells versus non-degranulated blue toluidine+ mast cells	-0.900	0.037
Area B		
mononuclear inflammatory cells versus c-kit+ mast cells	-0.086	0.872
mononuclear inflammatory cells versus tryptase+ mast cells	0.486	0.329
Mononuclear inflammatory cells versus total blue toluidine+ mast cells	-0.900	0.037
mononuclear inflammatory cells versus degranulated blue toluidine+ mast cells	-0.900	0.037
mononuclear inflammatory cells versus non-degranulated blue toluidine+ mast cells	-0.500	0.391

*Spearman’s correlation coefficient.

## Discussion

Mast cells have been implicated in the oral mucosa immune regulatory mechanism that controls the recruitment of inflammatory cells to the affected tissue.^17,24-26^ Given the nature of OLP and OLLC, immune cells have essential functions in etiopathogenesis of these lesions.^24,27^ OLP and OLLC have some similarities regarding clinical and histopathological features, although OLLC is a localized (contact) type IV hypersensitivity most often related to exposure to mercury.^2^ Classically, mast cells have been involved in allergic reactions and host defense to parasite infectious agents.^7^ Currently, the role of mast cells is well known in numerous physiological and pathological responses in autoimmune diseases and cancer.^13,14^ In this study, we identified a higher density of toluidine blue+, c-kit+, and tryptase+ mast cells in OLP and OLLC compared to HOM. However, we observed no difference in toluidine blue+, c-kit+, and tryptase+ mast cells between OLP and OLLC, rejecting our initial hypothesis. In addition, OLLC cases had a higher density of mononuclear inflammatory cells compared to OLP.

Our finding of elevated toluidine blue+ mast cells in patients with OLP and OLLC compared with HOM agrees with the results of other studies.^9,28,29^ Ramalingam et al.^9^ observed that OLP had the highest total count of mast cells compared with lichenoid reactions and HOM. Sharma et al.^28^also identified more toluidine blue+ mast cells in OLP and in oral lichenoid reaction than in HOM and no difference between OLP and oral lichenoid reaction. In contrast to our study, Juneja et al.^29^ observed an increased number of granulated toluidine blue+ mast cells in OLP. Another publication by Ghalayani et al.^17^ demonstrated that the expression levels of degranulated toluidine blue+ mast cells were significantly higher in oral lichenoid reactions than in OLP, a finding that we were unable to demonstrate in this study. Notably, degranulated toluidine blue+ mast cells reflect a state of activation and have been involved in the release of a spectrum of mediators that modulate other immune cells and the local microenvironment.^7,25^

Mast cell-specific surface marker, c-kit, is a protooncogene tyrosine kinase receptor (CD117) and has become a useful tool for identifying tissue mast cells.^30^ We found higher expression of c-kit in OLP and OLLC than in control. A similar result was found by Velez et al.^31^ who identified stronger expression of c-kit in pemphigus foliaceus relative to control biopsies. Żychowska et al.^32^ also identified c-kit in a cutaneous variant of lichen planus, but without differences in comparison to normal tissues. Likewise, a study by Mazreah et al.^33^ addressed expression levels of c-kit+ mast cells in periodontitis and found no difference in comparison to normal tissue. A study that analyzed the impact of c-kit+ mast cells in oral squamous cell carcinoma found that the presence of mast cells was associated with reduced disease recurrence.^26^ To address its potential function, c-kit signals mediate the normal growth and phenotypic differentiation of mast cells by regulating the production of their secretory granules.^25^ Curiously, we found that the staining pattern of c-kit closely resembled that of toluidine blue and tryptase, confirming the presence of mast cells and excluding a c-kit staining of true pluripotent stromal cells.

Tryptase is the major neutral protease present in human mast cell granules and is another specific marker for mast cells.^34^ We found increased numbers of tryptase+ mast cells in OLP and OLLC compared to normal tissue. Concerning the distribution of mast cells along the lamina propria, only in the analysis of tryptase, there was a difference in the presence of mast cells between the subepithelial band and the region below the subepithelial band in OLLC. Zhou et al.^24^ also described a greater number of tryptase+ mast cells in OLP than in normal control, especially in the superficial layer of the lamina propria. One explanation for the discrepancies between the location of mast cells in lamina propria may be related to the physiological distribution or the presence of a greater mononuclear infiltrate in the superficial layer of the lamina propria of these lesions.^25,34^ Given the nature of tryptase, dissolution of the basement membrane in OLP and OLLC may be mediated by mast cell proteases such as tryptase.^24^ Without denying or downplaying the importance of mast cells in both OLP and OLLC, we found no difference in tryptase+ mast cells expression between these lesions.

The current study showed that the density of mononuclear inflammatory cells in OLLC was higher, albeit not significantly different compared with OLP. Thornhill et al.^35^ investigated histopathological features that distinguished OLP from OLLC and found that a deep inflammatory infiltrate in some or all areas, a focal/perivascular infiltrate, and plasma cells or eosinophils favored the diagnosis of OLLC; however, the authors argued that the distinction between OLP and OLLC should not rely on histopathology alone. The density of mononuclear inflammatory cells may be related to the chronicity and form of treatment of the lesions – and not necessarily to the biology of OLP and OLLC. Notably, the presence of lymphoid follicles has been reported as a possible finding of OLLC.^2,27^

The interaction between/among immune components can determine the regulatory effect on OLP and OLLC by controlling oral insults. Curiously, we found a statistically strong correlation between mononuclear immune cells and mast cell density below the subepithelial band in OLP. In OLLC, on the other hand, the correlations between mononuclear immune cells and mast cell density along the lamina propria were negative. In this context, we speculated that the crosstalk between mast cells and other mononuclear immune cells is part of the pathogenesis of OLP, leading, at some point, to a greater dependence of mast cells compared to OLLC. In line with this finding, Telagi et al.^36^ showed that the number of mast cells increases with infiltration of immune cells in oral squamous cell carcinoma, suggesting a regulatory action on inflammatory responses, tumor cells, and extracellular matrix components.

The major limitation of this study was that we were unable to associate the staining findings with the clinical characteristics of the patients, such disease stage, presence of symptoms, and response to the treatment used. Although we used three different markers, some studies showed that mast cells have distinct phenotypes and may express markers other than those used here, which may have masked the detection of these cells.^25^ Virtually, all mast cells use c-kit for maturation and activation. It is also important to mention that tryptase and toluidine blue expression increases with mast cell activation.^25,34^

In conclusion, our data demonstrated that the etiopathogenesis process of OLP and OLLC modulates the expansion and degranulation of mast cells. We found that, quantitatively, mast cells in OLP are similar to those in OLLC. Although OLLC represent a type IV hypersensitivity reaction to mercury, weak and prolonged sensitization to mercury does not seem to mobilize a greater amount of mast cells or induce phenotypic changes that require further investigation. Further biochemical and molecular studies should be done with mast cells in oral lesions. It is clear that the biological distinction between OLP and OLLC may be helpful to understand the possibilities of malignant transformation of these two conditions. Finally, our findings demonstrated that toluidine blue, c-kit, and tryptase were sensitive in the identification of mast cells in the assessed tissues.
